# Lentivirus Live Cell Array for Quantitative Assessment of Gene and Pathway Activation during Myogenic Differentiation of Mesenchymal Stem Cells

**DOI:** 10.1371/journal.pone.0141365

**Published:** 2015-10-27

**Authors:** Janhavi Moharil, Pedro Lei, Jun Tian, Daniel P. Gaile, Stelios T. Andreadis

**Affiliations:** 1 Bioengineering Laboratory, Department of Chemical and Biological Engineering, University at Buffalo, State University of New York, 908 Furnas Hall, Amherst, NY 14260–4200, United States of America; 2 Department of Biostatistics, University at Buffalo, State University of New York, Kimball, Buffalo, NY 14214–3000, United States of America; 3 Department of Biomedical Engineering, University at Buffalo, The State University of New York, Amherst, NY 14260–4200, United States of America; 4 Center of Excellence in Bioinformatics and Life Sciences, Buffalo, NY 14203, United States of America; National University of Ireland, Galway (NUI Galway), IRELAND

## Abstract

Stem cell differentiation involves multiple cascades of transcriptional regulation that govern the cell fate. To study the real-time dynamics of this complex process, quantitative and high throughput live cell assays are required. Herein, we developed a lentiviral library of promoters and transcription factor binding sites to quantitatively capture the gene expression dynamics over a period of several days during myogenic differentiation of human mesenchymal stem cells (MSCs) harvested from two different anatomic locations, bone marrow and hair follicle. Our results enabled us to monitor the sequential activation of signaling pathways and myogenic gene promoters at various stages of differentiation. In conjunction with chemical inhibitors, the lentiviral array (LVA) results also revealed the relative contribution of key signaling pathways that regulate the myogenic differentiation. Our study demonstrates the potential of LVA to monitor the dynamics of gene and pathway activation during MSC differentiation as well as serve as a platform for discovery of novel molecules, genes and pathways that promote or inhibit complex biological processes.

## Introduction

Stem cell differentiation involves exogenous signals that activate signaling pathways leading to transcriptional activation of lineage specific genes. Such signals include soluble factor [1–4], growth factors [5–8], extracellular matrix components and mechanical forces such as those exerted by the substrate supporting the cells [4, 9]. These signals activate biochemical pathways leading to transcriptional changes dictating stem cell lineage specification over a period of days to weeks. To understand the collective dynamics of the process, it is important to capture the dynamics of gene and pathway activation for a broad array of genes and pathways that may be involved during stem cell differentiation. In turn, this requires development of large-scale live cell assays to capture the dynamics in real time and in a quantitative manner.

Most high-throughput genomic and proteomic methods available today require cell destruction and therefore, they are not easily amenable to repeated dynamic interrogation. On the other hand, reporter based assays can provide quantitative and real-time measurements of gene and pathway activation [10–14]. Reporter assays make use of reporter proteins such as luciferase or fluorescence proteins (ZsGreen, DsRed) to measure the activity of a gene promoter (Pr) or a transcription factor (TF) binding site (Response Element, RE). In this context, Pr activity reflects transcription of the corresponding gene, while RE activity reflects activation of the upstream signaling pathway(s) leading to TF activation (e.g. phosphorylation) and ensuing transcription of the reporter gene.

However, stem cells and in particular MSCs are notoriously difficult to transfect, thereby requiring long-term drug selection during which MSCs senesce, limiting their proliferation and multi-lineage differentiation capacity [15–17]. Even for cells that are easily transfectable, the transient nature of transfection makes it difficult to follow them for the time required to complete lineage specification. Therefore, development of novel strategies that enable high throughput, real-time and quantitative measurements of pathway activation would greatly facilitate the understanding of stem cell lineage commitment as well as other complex biological processes.

To this end, our laboratory designed a novel lentiviral dual promoter vector (LVDP) carrying two independent gene cassettes [18]. In the first, the Pr/RE of interest drives expression of a reporter protein (e.g. ZsGreen); and in the second, a constitutive promoter (e.g. human phosphoglycerate kinase promoter, hPGK) drives expression of a second reporter (e.g. DsRed) that is used to measure transduction efficiency and for data normalization [18, 19]. We also developed novel methods to immobilize lentiviral (LV) particles on surfaces including hydrogels [20, 21] that facilitated the development of the LVA to measure the activity of many Pr/RE in a high throughput manner. The LVA technique was demonstrated to measure the activity of several Pr/RE participating in the inflammatory response [19] and more recently in MSC differentiation into fat, bone and cartilage [22]. In this study, we employed the LVA to quantitatively capture gene expression dynamics over a period of several days during differentiation of MSCs into smooth muscle cells (SMCs) using a set of 27 Pr/RE. The Pr/RE dynamics enabled us to identify differences between MSCs from different anatomic locations and in combination with small chemical inhibitors, to determine the relative contribution of key signaling pathways during MSC commitment to the myogenic lineage.

## Materials and Methods

### Cell culture

293T/17 cells (ATCC, Manassas, VA) were cultured in Dulbecco’s Modified Eagle Medium (DMEM; GIBCO BRL, Grand Island, NY) supplemented with 10% (v/v) Fetal Bovine Serum (FBS; GIBCO) and 1% (v/v) Antibiotic-Antimycotic (Anti-Anti; GIBCO). Human Hair Follicle derived Mesenchymal Stem Cells (hHF-MSCs) from a 73 year old male donor were isolated and characterized for differentiation potential as described previously [16, 23] and human Bone Marrow derived Mesenchymal Stem Cells (hBM-MSCs, 29 year old male; Stem Cell Technologies, Vancouver, Canada) were cultured in growth medium (GM): DMEM supplemented with 10% (v/v) Mesenchymal Stem Cell qualified Fetal Bovine Serum (MSC-FBS; GIBCO), 1% (v/v) Anti-Anti and 1 ng/ml basic Fibroblast Growth Factor (bFGF; Biolegend, San Diego, CA). Cells were induced to myogenic differentiation using myogenic differentiation medium (DM): DMEM supplemented with 10% (v/v) MSC-FBS and 1% (v/v) Anti-Anti + 10 ng/ml TGF-β1 (Biolegend) + 30 μg/ml Heparin (APP Pharmaceuticals, LLC, Schaumburg, IL).

### Flow Cytometry

hBM-MSCs transduced with LVDP carrying the ACTA2 promoter were cultured in 24 well tissue culture treated plates in GM or DMEM supplemented with 10% (v/v) MSC-FBS + 1% (v/v) Anti-Anti + potential myogenic inducers: TGF-β1 (0, 1, 2, 5, 10, 20 ng/ml) or TGF-β1 (10 ng/ml) + (30 μg/ml Heparin or 30 μM Ascorbic Acid (AA; Sigma, St. Louis, MO) or 2 μM Insulin (I; Sigma)) for 2 days. Afterwards, cells were washed once in PBS and detached from the surface using 0.25% Trypsin/EDTA (GIBCO). Both red and green fluorescent intensities were measured using flow cytometry (FACSCalibur; Becton Dickinson, San Jose, CA).

### Immunostaining

hBM-MSCs were cultured in GM or DM for 7 days. Cells were immunostained for the presence of smooth muscle cell specific markers as described previously [24]. The following primary antibodies diluted in 5% (v/v) goat serum were used: mouse monoclonal anti-human αSMA (1:100 dilution; Serotec, Raleigh, NC), mouse monoclonal anti-human CNN1 (1:100 dilution; Santa Cruz Biotechnology, Dallas, TX), and rabbit monoclonal anti-human MYH11 (1:100 dilution; Biomedical Technologies Inc., Stoughton, MA). Cells stained with secondary antibody only, which did not show any fluorescence signal, served as a negative control.

### Western Blot

hHF-MSCs were cultured in DM in the presence of the indicated inhibitors for 7 days. Cells cultured in GM and DM served as control. Afterwards, cells were lysed, and the expression level of αSMA in the lysates was detected with a mouse monoclonal anti-human αSMA antibody (1:2000 dilution in 5% (v/v) non-fat milk) by western blot as described previously [25]. Rabbit monoclonal anti-GAPDH antibody (14C10; Cell Signaling Technology, Danvers, MA) was used as loading control. Images were analyzed using CellProfiler (BROAD Institute, www.cellprofiler.org). Quantified band intensities were averaged across replicate experiments (n = 3) and expressed as mean ± standard deviation.

### Vector Construction

Promoters or transcriptional response elements of genes that are potentially involved in myogenic differentiation (**S1 Table**) were cloned in the LVDP vector that was previously developed in our laboratory [19]. The Pr/RE-of-interest drives the expression of ZsGreen (ZsG; t_1/2_ = 120 hrs), while the human PGK promoter drives expression of DsRed-Express2 (DRE2; t_1/2_ = 24 hrs). Primers and oligos used for cloning promoters and transcriptional response elements, respectively, are listed in **S1 Table**.

The vector pCS_SMAR8_pA1_DRE2_hPGK_cHS4_Tactb_SPA_ZsG_MCS was used for promoter cloning via the ClaI/AgeI or EcoRI/AgeI restriction sites, while the vectors pCS_SMAR8_pA1_DRE2_hPGK_cHS4_Tactb_SPA_ZsG_RE-NFKB_MCS or pCS_ SMAR8_pA1_DRE2_hPGK_cHS4_Tactb_SPA_ZsG_RE-NFKB_MCS2 was used for cloning of transcriptional response elements via the restriction sites BstBI/HpaI and BstBI/BsiWI, respectively (**S1 Table**). Lentiviral library carrying different promoters and transcriptional response elements was produced by the standard calcium phosphate precipitation method in 293T/17 cells. The lentivirus titers were estimated to be 10^7^–10^8^ IFU/ml by using 293T/17 cells.

### Lentiviral Array

MSCs were seeded in tissue culture treated optical bottom 384 well plate (Thermo Scientific, Rochester, NY) at a density of 1000–2000 cells per well. On the next day, the cells were transduced with the lentiviral library in the presence of 8 μg/ml Polybrene in quadruplicates for overnight. Growth medium was replenished the next day. In 72 hours post transduction when the cells started to express the red fluorescence protein, an indication of transduction efficiency, some cells were treated with DM. Cells that were still cultured in GM were used as control. For inhibitor screening, cells were treated with DM in the presence or absence of chemical inhibitors (**S2 Table**). The Pr/RE activity was monitored by continuous imaging (**see** **Image Acquisition & Quantification**). The differentiation and growth media were replenished every two days.

### Image Acquisition & Quantification

Red and green fluorescence images for each sample were captured with an automated fluorescence microscope (Axio Observer Z1, Carl Zeiss Inc, Thornwood, NY) at 5x magnification for same exposure times (100–150 ms). Images were acquired at regular intervals up to 6–7 days after treatment. Intensities of all images were quantified by the image analysis software CellProfiler. Pipetting errors, illumination variation, effects of media changes and other unknown well-to-well differences caused background variations necessitating the calculation of local intensity thresholds instead of a single global threshold. Mixture of Gaussian (MoG) method was used for thresholding in CellProfiler to identify fluorescent cells in each image. The size range for identifying cells was optimized to (10,600) pixel units. The fraction of image covered by the cells was fixed at 0.9. A threshold correction factor of 1.03 was set to identify the true objects and eliminate the false positives resulting from illumination variation. Finally the total integrated intensity of the identified objects for each fluorescent channel in each image was determined.

### Data Normalization

For all dynamic LV array experiments, a data driven approach was used for normalization. Consider a time profile of GFI and corresponding RFI values as (*g*_*ghij1*_, *r*_*ghij1*_), (*g*_*ghij2*_, *r*_*ghij2*_),…………,(*g*_*ghijK*_, *r*_*ghijK*_) where *g = 1*,*2*,*……*,*G* (replicate experiments), *h = 1*,*2*,*……*,*H* (Pr/RE used), *i = 1*,*2*,*……*,*I* (experimental conditions), *j = 1*,*2*,*……*,*J* (replicate wells within an experiment g) and *k = 1*,*2*,*……*,*K* (time points) (**see S3 Table for parameter values**). Next, a weighted average of RFI for each time profile was calculated. The weights were determined from a beta distribution on an interval (0, 1) using a nonlinear minimization routine to minimize the median absolute standard deviation of the normalized intensity for each Pr/RE from its LOESS fit. The optimization was done across all replicate datasets for each cell type. The normalized intensities were calculated by dividing the GFI with the corresponding weighted average of RFI. Each time profile was further normalized by the corresponding intensities at time *t = 0* and the final intensities are denoted as Normalized Intensity (*NI*). Data normalization was performed in R programming language.

### Re-Scaling data from inhibitor screening

For comparison of Pr/RE activity in cells treated with DM + chemical inhibitors to cells cultured in DM (positive control) and in GM (negative control), the data were scaled as in Eq 1 such that the *NI* in all 3 culture conditions on day 1 (*t = 0 hours*, *k = 1*) is 0 and *NI* in DM on day 7 (*t = 168 hours*, *k = K = 15*) is 1.

In each experiment g, for each Pr/RE, h,

$$NI_{h,i,j,k}=\frac{NI_{h,i,j,k}-NI_{h,min}}{NI_{h,max}-NI_{h,min}}$$ (1)

Where ${NI}_{h,max}\;=\sum_{j=1}^{J}\;{NI}_{h,DM,j,K}/J$ and ${NI}_{h,min}\;=\;\sum_{i=1}^{I}\sum_{j=1}^{J}\;{NI}_{h,i,j,1}/J$*I

### Data processing for heatmaps

The standardized fold change (Welch’s *t*-statistic) in DM with respect to GM at each time point was calculated as,

$$t_{h,k}=\frac{NI_{GM,k}-NI_{DM,k}}{\sqrt{\frac{s_{DM,k}^{2}}{J_{DM,k}}+\frac{s_{GM,k}^{2}}{J_{GM,k}}}}$$ (2)

Heatmaps of the standardized fold change were plotted in MATLAB.

### Statistical Analysis

For differentiation medium optimization and western blot quantification, data from triplicates were averaged. Statistical analysis of data was determined by using a two-tailed Student’s t-test (α = 0.05) in Microsoft Excel (Microsoft, Redwood, CA). The sample size was *n* = 3. For all dynamic LV array experiments, we used a growth curve analysis approach [26] that fits a linear mixed effects model to compare the effects of time and experimental condition on Pr/RE responses. The time course was modeled with a fourth order polynomial. The effects of experimental conditions (GM, DM, DM + inhibitors) were modeled as fixed effects on all time terms while the random effects were modeled by replicate effect on all time terms. The significance of each parameter on the model fit was evaluated by log likelihood. The analysis was done in R programming language using the statistical package ‘lme4’. The pairwise statistical significance over individual time points was evaluated by a two-tailed Student’s t-test (α = 0.05) in Microsoft Excel.

## Results

### Normalized Pr/RE activity is independent of gene transfer efficiency

Previously we reported the design of a novel LVDP vector that enables independent gene expression from two independent promoters or regulatory response elements. Transcriptional regulatory units–poly(A) tail, terminator and insulator sequences–were inserted between two expression cassettes to eliminate promoter interference, resulting in gene expression levels comparable to vectors carrying a single transcription unit [18]. Herein we used the LVDP to monitor transcriptional activity during differentiation of MSCs along the smooth muscle cell lineage. To this end, literature-reported promoters of SMC genes and a number of transcription factor consensus-binding sites were cloned into LVDP in order to capture signaling pathways that may be involved during MSC differentiation. The entire list of Pr/RE that were cloned into the LVDP is shown in **Table 1**.

**Table 1:** List of Promoters and Response Elements cloned into LVDP.

Promoters	Response Elements	
SM22-Pr	MAPK/ERK-RE	Nanog-RE
SMTNB-Pr	CArG-RE	STAT3-RE
rMYH11-Pr (rabbit)	CArGA-RE	p53-RE
ACTA2-Pr	EGR1-RE	SMAD2/3-RE
DES-Pr	KLF4-RE	SMAD4-RE
CSRP2-Pr	MEF2-RE	SMAD7-RE
MKL2-Pr	SP1-RE	SMAD1/5/8-RE
ACTB-Pr	ATF6-RE	Notch-RE
MKL1-Pr	HIF1-RE	
PITX2-Pr		

In each LVDP, the Pr/RE of interest drives expression of one reporter (e.g. ZsGreen) and a constitutive promoter (e.g. hPGK) drives expression of a second reporter (e.g. DsRed-Express2, DRE2), enabling signal normalization that is necessary to render the fluorescence intensity measurements independent of the gene transfer efficiency. Specifically, sample-to-sample variation in green fluorescence intensity (GFI) may reflect differences in the fraction of transduced cells or the number of proviruses per cell, and not necessarily the response of the Pr/RE to the differentiation-inducing signals. In principle, this problem could be alleviated by normalizing the GFI by the signal of another gene e.g. red fluorescence intensity (RFI) of DRE2, which is expressed under an independent and constitutive promoter (hPGK).

**Fig 1:**
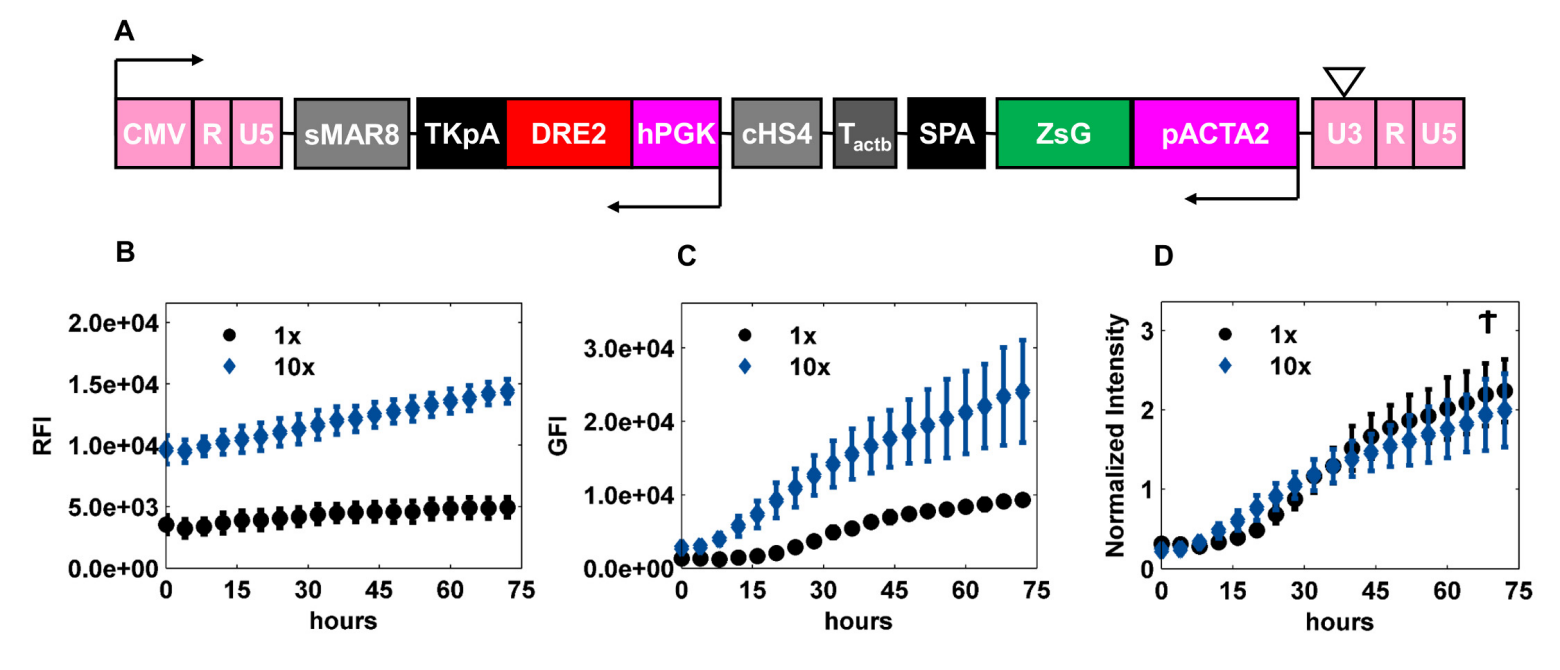
Normalized Promoter Activity is independent of gene copies per cell. **(A)** Schematic of LVDP-pACTA2-ZsGreen. CMV–promoter sequence from cytomegalovirus; sMAR8 –synthetic MAR sequence 8; TKpA–thymidine kinase polyA from herpes simplex virus; DRE2 –DsRed Express 2; hPGK–human phosphoglycerate kinase promoter; cHS4 –chicken hypersensitive site 4; T_actb_−a G-rich sequence from the extension of β-actin gene; SPA–synthetic poly A; ZsG–ZsGreen; pACTA2 –α smooth muscle actin promoter; 5’ LTR: CMV + R + U5; 3’LTR: U3 + R +U5 **(B-D)** hBM-MSCs were transduced with LVDP-pACTA2-ZsGreen at two different concentrations that differed by 10-fold (1X and 10X). Both red and green fluorescence intensity were measured upon induction to myogenic differentiation. **(B)** Red Fluorescence Intensity (RFI), **(C)** Green Fluorescence Intensity (GFI), and **(D)** Normalized Intensity were plotted against time. Normalized intensity was obtained by dividing GFI by a weighted average of RFI over time, where the weights were determined from a beta distribution (see Materials and Methods) such that the median absolute standard deviation was minimized from the LOESS fit. Data shown are representative of at least three experiments performed with similar results. Ϯ p-value > 0.05.

To examine whether the normalized fluorescence intensity was independent of the transduction efficiency, we chose the promoter of alpha smooth muscle actin (ACTA2)–an early marker of the smooth muscle cell lineage—and cloned it upstream of ZsGreen (LVDP-pACTA2-ZsGreen, **Fig 1A**). Subsequently LVDP-pACTA2-ZsGreen was used to transduce hBM-MSCs at two concentrations, 1X and 10X. Cells were then coaxed to differentiate into SMC, and GFI and RFI were measured every 4 hr for 3 days by fluorescence microscopy. As expected, cells transduced with the high concentration virus (10X) showed increased RFI at all time-points, reflecting higher transduction efficiency compared to cells transduced with lower virus concentration (1X) **(Fig 1B)**. GFI was low at early time points in both cases, possibly because the ACTA2 promoter was still inactive. As the cells differentiated, the ACTA2 promoter was activated and GFI reached higher levels over a period of time with the intensity being higher in cells that were transduced with the higher virus concentration **(Fig 1C)**. We then normalized the GFI for each virus concentration by a weighted average of the corresponding RFI over all time points. The weights were determined from a beta distribution (see Materials & Methods) such that the median absolute standard deviation of the normalized intensities across the replicates was minimized from its LOESS fit. As expected, the Normalized Intensity (NI) reached similar levels for both virus concentrations **(Fig 1D)**. These results thus indicated that the intrinsic promoter activity was independent of transduction efficiency, thereby enabling quantitative measurements of gene/pathway activation during differentiation.

### Optimization of myogenic differentiation medium

Since SMC differentiation is accompanied by enhanced protein expression of alpha smooth muscle actin (αSMA), we used the ACTA2 promoter activity to optimize the differentiation medium. To this end, hBM-MSCs were transduced with the LVDP-pACTA2-ZsGreen. Cells were either cultured in growth medium (GM: 1 ng/ml bFGF, no TGF-β1), which served as a control or exposed to different concentrations of TGF-β1 (0, 1, 2, 5, 10, and 20 ng/ml), a potent multifunctional cytokine that is thought to regulate a number of the cellular events underlying the development of vascular lesions, including SMC differentiation [27–32]. TGF-β1 has been shown to stimulate expression of αSMA, Myosin Heavy Chain (MYH11) and Transgelin (SM22) in SMCs that have undergone partial dedifferentiation (or modulation) in culture [31–35]. Because SMC differentiation is characterized by the upregulation of these and other smooth muscle specific genes, TGF-β1 is thus believed to be a critical factor for driving MSC differentiation into SMCs. Moreover, several studies have reported that a synergistic effect of TGF-β1 with other chemical factors enhances the expression of smooth muscle specific genes [2, 3, 36, 37].

**Fig 2:**
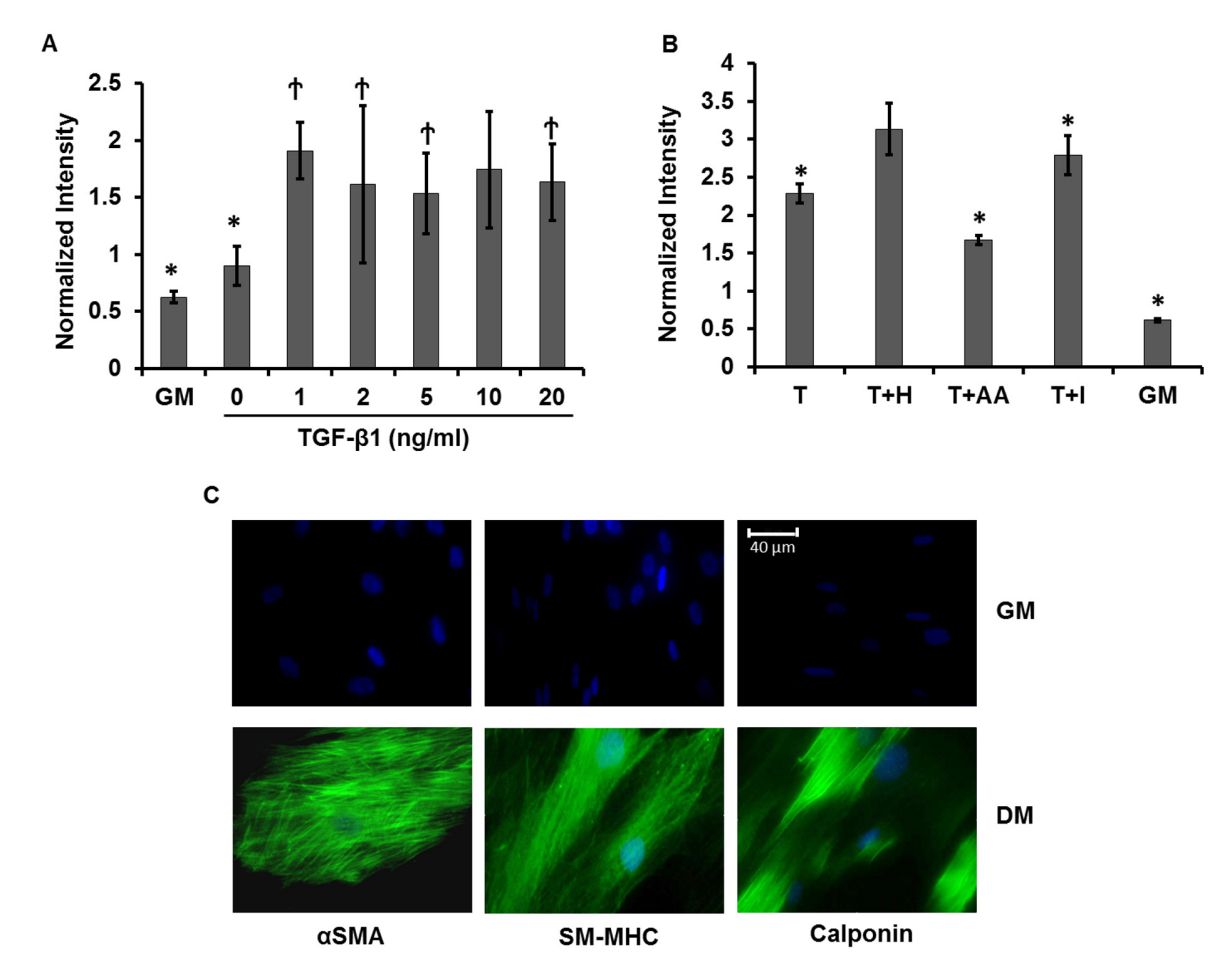
Optimal conditions for myogenic differentiation. Optimized myogenic differentiation medium was determined by treating LVDP-pACTA2-ZsGreen transduced hBM-MSCs with **(A)** varying concentrations of TGF-β1 (0, 1, 2, 5, 10, and 20 ng/ml) or **(B)** a combination of 10 ng/ml TGF-β1 (T) and one of the following soluble factors: 30 μg/ml Heparin (H), 30 μM Ascorbic Acid (AA), or 2 μM Insulin (I). Red and green fluorescence intensities were measured by flow cytometry after 2 days and normalized intensity was shown. **(A)** * denotes p < 0.05 as compared to 10 ng/ml TGF-β1, ^Ϯ^ denotes p > 0.05 as compared to 10 ng/ml TGF-β1. **(B)** * denotes p < 0.05 as compared to T+H. **(C)** Immunostaining for SMC proteins (αSMA, smooth muscle myosin heavy chain (SM-MHC) and Calponin) under GM or the optimized differentiation medium (DM, T+H) for 7 days. Cell nuclei were counterstained with the Hoechst 33342. Images are representative of three independent experiments. Scale bar: 40 μm.

After 2 days of treatment, fluorescence intensity was measured by flow cytometry. The normalized ACTA2 promoter activity (GFI/RFI) increased in the presence of TGF-β1, reaching 2–3 fold higher than in GM at concentrations between 2–20 ng/ml of TGF-β1 **(Fig 2A)**.

We also investigated whether addition of other factors that were previously reported to promote SMC differentiation e.g. ascorbic acid [38], insulin [39], and heparin [40, 41] could further increase ACTA2 promoter activity. As shown in **Fig 2B** the combination of TGF-β1 and heparin consistently yielded the highest ACTA2 promoter activity and therefore, it was used in the differentiation medium (DM) to coax MSC differentiation into SMC in all subsequent experiments.

### TGF-β1 and heparin induced expression of SMC proteins in hBM-MSCs

Next we assessed whether the ACTA2 promoter activity that was induced by DM correlated with protein expression. To this end, hBM-MSCs were cultured for 7 days in GM or DM and the presence of SMC specific proteins was assessed by immunostaining **(Fig 2C)**. While αSMA was weakly expressed in GM, high expression of well-organized and brightly stained actin filaments was induced after 7 days in DM. Additionally, other SMC proteins such as Calponin and the late differentiation marker, smooth muscle myosin heavy chain were also significantly enhanced and displayed fibrillar organization in DM **(Fig 2C)**. Collectively, these data indicated that increased ACTA2 promoter activity correlated well with the differentiation of hBM-MSC into SMC phenotype as evidenced by the elevated levels and fibrillar organization of early, intermediate and late SMC proteins.

### Dynamics of Pr/RE activation during myogenic differentiation of MSCs

**Fig 3:**
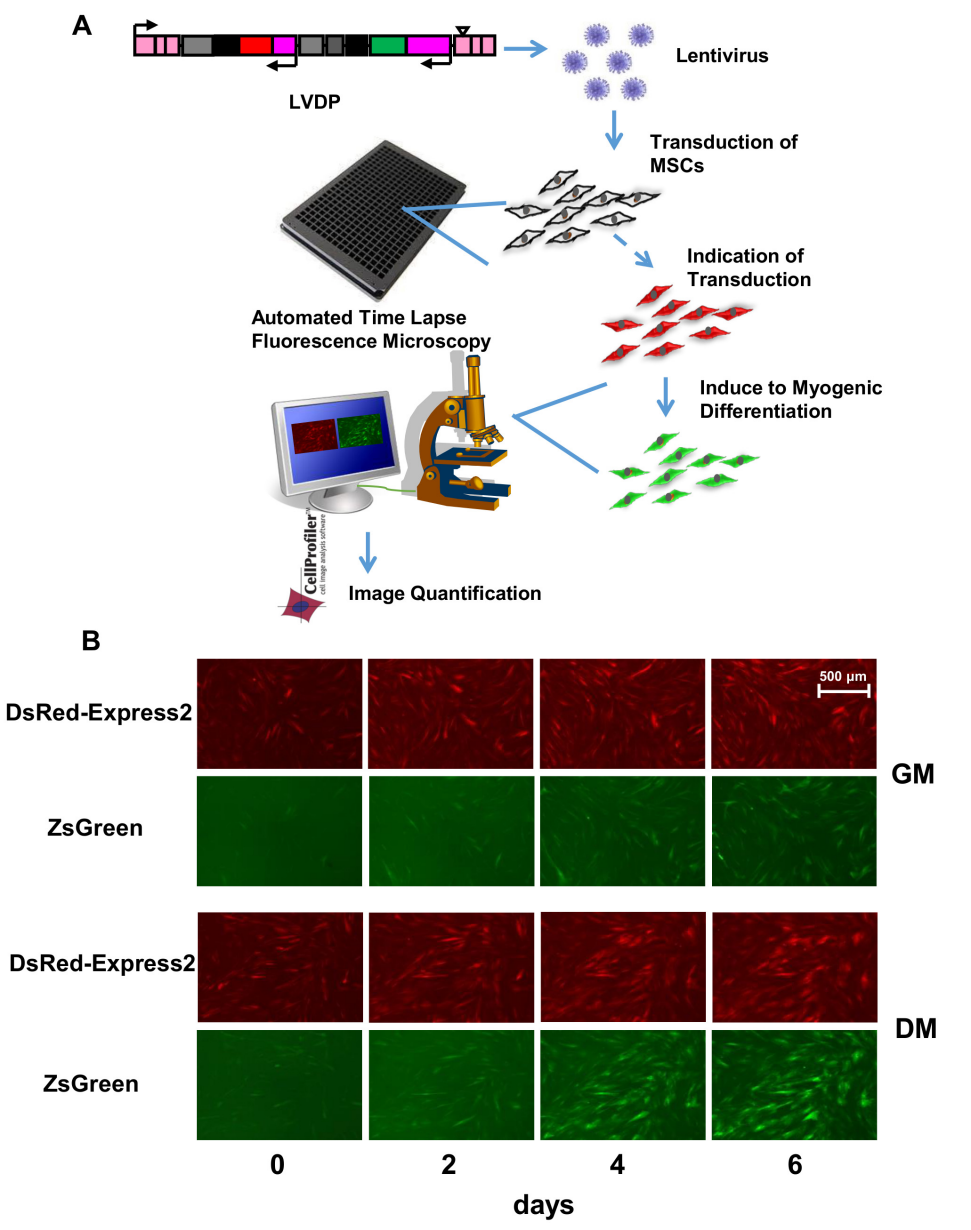
High throughput monitoring of Pr/RE activity. **(A) Schematics of Lentiviral Array. (A)** Pr/RE of genes potentially involved in myogenic differentiation were cloned in the LVDP. Recombinant lentiviruses were generated and used to transduce MSCs in 384 well plates. Upon successful transduction as evidenced by red fluorescence expression, MSCs were induced to myogenic differentiation. Red and green fluorescence were imaged every 8 hr using fluorescence microscope with automated stage for 6–7 days. GFI and RFI for each image were quantified by CellProfiler. **(B)** Representative red **(row 1 and 3)** and green **(row 2 and 4)** fluorescence images of hBM-MSCs transduced with LVDP-pACTA2-ZsGreen under growth **(GM, row 1 and 2)** or differentiation **(DM, row 3 and 4)** conditions at the indicated time (0, 2, 4 and 6 days) of treatment with DM. Scale bar: 500 μm.

We then employed the LVDP to create a lentiviral array to monitor active pathways in hBM-MSCs and hHF-MSCs during myogenic differentiation. First, we generated a library of lentiviruses each carrying a Pr/RE that may be associated with SMC phenotype. In total we generated LVDP with 10 Pr and 17 transcriptional RE representing several SMC markers, constituents of cytoskeleton apparatus and signaling pathways including the TGF-β, p53, mitogen-activated protein kinase (MAPK), Notch, JAK/STAT, activating transcription factor 6 (ATF6), and hypoxia related pathways **(Table 1)**. Subsequently MSCs were transduced with this LV library in 384 well plates and 3 days later the cells were coaxed to differentiate into SMC. MSCs that were transduced but cultured in GM for the same time period served as control **(Fig 3A)**.

Red and green fluorescence intensity were monitored under growth and differentiation conditions at regular time intervals, i.e. every 8 hr for 6–7 days using fluorescence microscopy. Red and green fluorescent images from cells transduced with LVDP-ACTA2-ZsGreen at the indicated time points are shown in **Fig 3B**. Fluorescence intensities were quantified using CellProfiler. A weighted average normalization of GFI by RFI across experimental replicates was done to obtain dynamic profiles of normalized intensity (NI) for the 27 Pr/RE in GM and DM for both hBM-MSCs and hHF-MSCs (**see** Materials & Methods).

**Fig 4:**
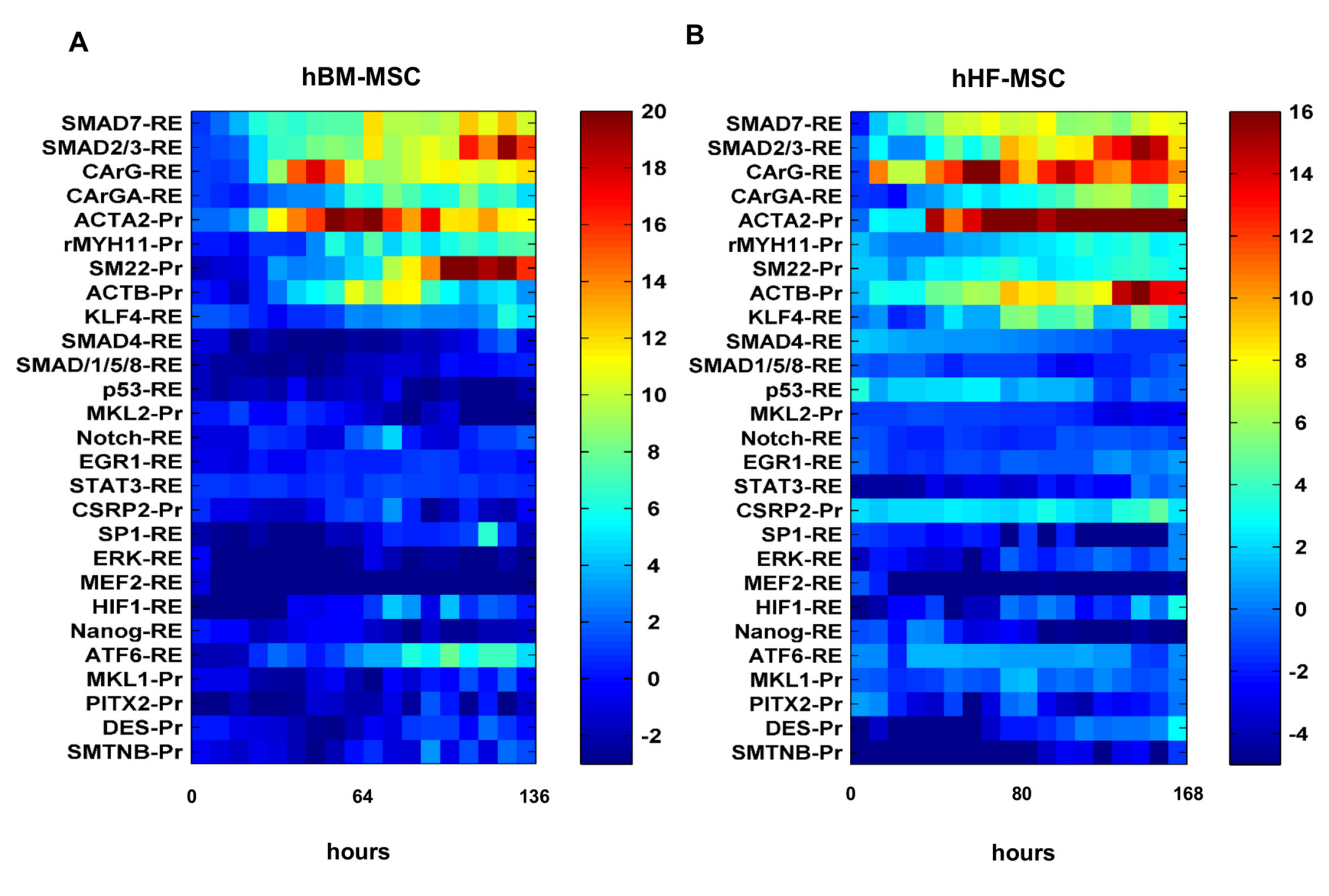
Heatmaps of Pr/RE responses. Standardized fold change (Welch’s *t-*statistic) of normalized intensity in DM over GM for **(A)** hBM-MSC or **(B)** hHF-MSC was plotted as a function of time for 27 Pr/RE. The color bar represents the standardized fold change on a scale of (**A)** -3 to +20 and (**B)** -5 to +16. The color gradient ranges from blue (no significant change) to red (highly up-regulated).

Heatmaps of all Pr/RE activities in DM with respect to GM for both the MSCs are shown in **Fig 4**. Nine constructs (5 RE and 4 Pr) had statistically significant activities in DM as compared to GM in both MSCs. These included members of the TGF-β signaling pathway, SMC specific proteins, transcription factors involved in myogenic differentiation and constituents of the cytoskeleton apparatus. Fold change for each Pr and RE was calculated as the mean over the last four time points of NI in DM (NI_DM_) divided by NI in GM (NI_GM_), averaged across all experiments and denoted by NI_DM/GM_ (**S4 Table**). The statistical significance of the entire curve was calculated by the growth curve analysis approach [26], a multilevel regression technique for time course analysis (**see** Materials & Methods). Also, a pairwise comparison was done at each time point by a two-tailed Student’s *t*-test. The time of activation of Pr/RE was identified as the time at and beyond which Pr/RE yielded a statistically significant response in DM as compared to GM. The average time of activation of Pr/RE in both the cells types is available in **S5 Table**.

**Fig 5:**
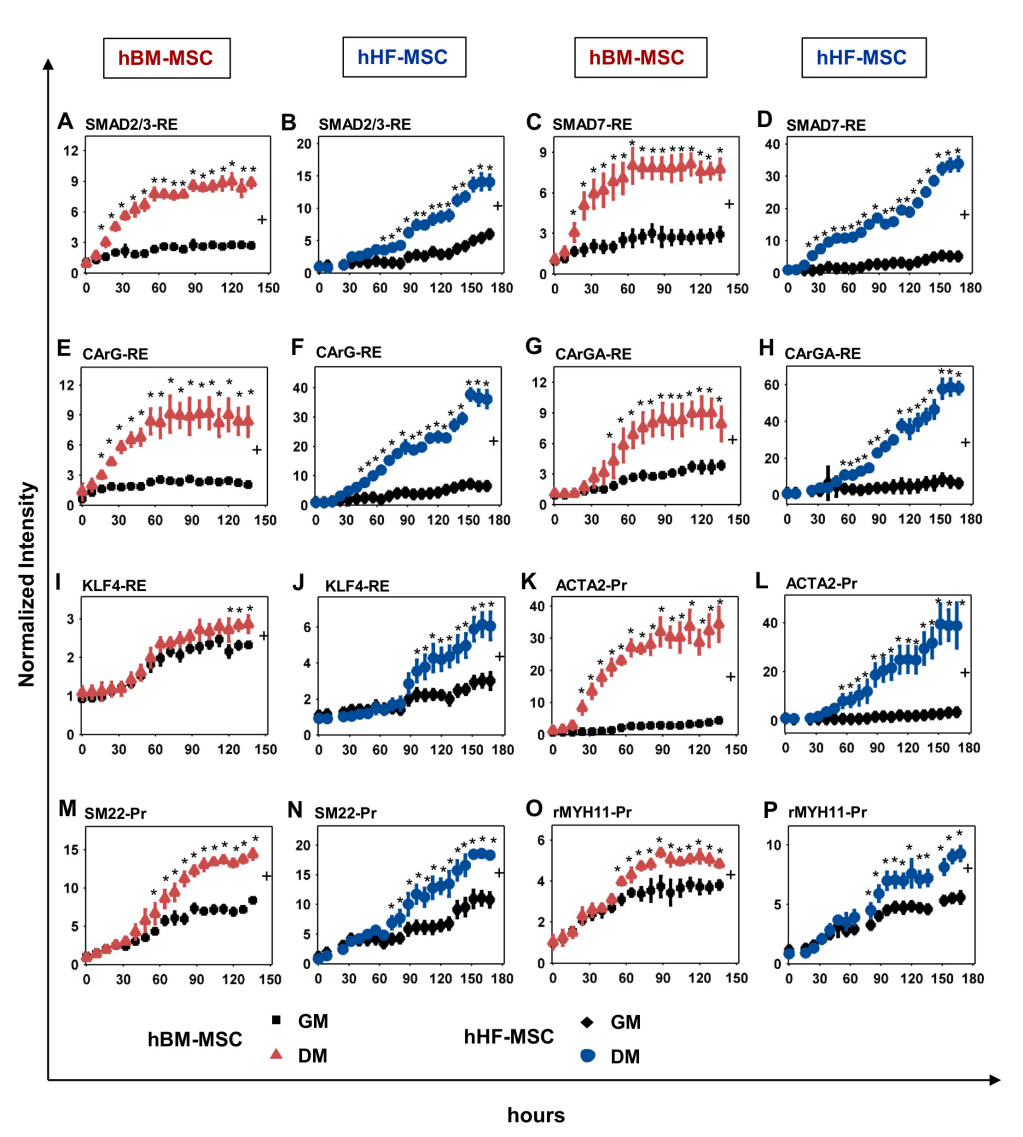
Dynamic response of Pr/RE in MSCs during Myogenic Differentiation. **(A, C, E, G, I, K, M, O)** hBM-MSCs or **(B, D, F, H, J, L, N, P)** hHF-MSCs were transduced with the LV library of 27 Pr/RE and coaxed to differentiate into SMCs. The activities of the **(A, B)** SMAD2/3-RE; **(C, D)** SMAD7-RE; **(E, F)** CArG-RE; **(G, H)** CArGA-RE; **(I, J)** KLF4-RE; **(K, L)** ACTA2-Pr; **(M, N)** SM22-Pr; and **(O, P)** rMYH11-Pr were determined by fluorescence microscopy and the normalized intensity was plotted as a function of time. MSCs cultured in GM served as a control. * indicates p < 0.05 between DM and GM as determined by Student’s two-tailed *t*-test at individual time points. + indicates statistical significance of the Pr/RE activities under DM vs GM evaluated over entire curve by growth curve analysis (p < 0.05).

The Smad signaling pathway was the first to be activated in DM as indicated by the rapid response of Smad REs in both cell types. In hBM-MSCs Smad2/3-RE activity increased rapidly after 8 hr until it reached a steady state of about 3-fold (NI_DM/GM_ = 3.17 ± 0.04) **(Fig 5A)**. Interestingly, Smad2/3-RE was rather slow to respond in hHF-MSCs up to day 2 but gradually increased to ~2.5-fold compared to GM (NI_DM/GM_ = 2.7± 0.42) **(Fig 5B)**. Smad7-RE also showed a quick response (~16 hr) and reached >2-fold in hBM-MSCs (NI_DM/GM_ = 2.59 ± 0.31) and significantly higher in hHF-MSCs (NI_DM/GM_ = 5.14 ± 2.3) **(Fig 5C and 5D)**. On the other hand, SMAD4-RE (co-smad) and SMAD1/5/8-RE did not respond to DM treatment in either cell type (**Fig 4**).

In addition to phosphorylating Smads, treatment with TGF-β is known to activate serum response factor (SRF), which binds to the CArG response element, a serum response element (SRE) found in the promoter region of almost all smooth muscle specific genes [42]. Indeed, we observed a rapid upregulation of CArG-RE (t_r_ = 24–30 hr), which increased significantly in both cell types (NI_DM/GM_ = 3.75 ± 0.005 in hBM-MSCs and 5.19 ± 0.56 in hHF-MSCs) **(Fig 5E and 5F)**. On the other hand, CArGA-RE, which is present in the promoter of ACTA2 [43], exhibited slower response in both cell types, and reached fold increase that was significantly higher in hHF-MSCs (NI_DM/GM_ = 5.47 ± 3.40) as compared to hBM-MSCs (NI_DM/GM_ = 2.37 ± 0.003) **(Fig 5G and 5H)**. The RE of the myogenic differentiation inhibitor Klf4 (KLF4-RE) did not respond in the first 4 days and subsequently it increased in hHF-MSCs (NI_DM/GM_ = 1.76 ± 0.52) but only marginally in hBM-MSCs (NI_DM/GM_ = 1.2 ± 0.0005) **(Fig 5I and 5J)**.

Interestingly, the activation of the TGF-β1 pathway (Smad-REs) and CArG-RE was followed by increased activity of several smooth muscle gene promoters. The promoter activity of the early myogenic marker, αSMA (ACTA2-Pr), was the quickest to respond (~ 30 hr) and increased significantly by 7-fold in hBM-MSCs (NI_DM/GM_ = 7.19 ± 2.68) and by 12-fold in hHF-MSCs (NI_DM/GM_ = 12.4 ± 1.4) **(Fig 5K and 5L)**. The intermediate marker, SM22 had strong basal expression and increased over time in GM and DM in both cell types. Under DM, it showed a moderate response after day 2 in both hBM-MSCs (NI_DM/GM_ = 1.77 ± 0.13) and hHF-MSCs (NI_DM/GM_ = 1.62 ± 0.26) **(Fig 5M and 5N)**. Finally, the late myogenic marker, myosin heavy chain (rMYH11) was the last to respond and increased only slightly in hBM-MSCs (NI_DM/GM_ = 1.34 ± 0.004) and in hHF-MSCs (NI_DM/GM_ = 1.4 ± 0.17) **(Fig 5O and 5P)**.

Promoters of genes encoding for cytoskeletal proteins also increased. Specifically, the ACTB (β-actin) promoter increased significantly by 3.45 ± 0.12 fold in hBM-MSCs and 4.62 ± 0.53 fold in hHF-MSCs above the basal values **(S4 Table)**. However, the promoters of other transcription factors implicated in myogenic differentiation such as Paired like homeodomain transcription factor 2 (PITX2), Sp1 transcription factor (SP1), Myocardin-related transcription factor A (MRTFA, MKL1) and Myocardin-related transcription factor B (MRTFB, MKL2) did not respond to DM stimulation in either cell type. Also most other REs representing signaling pathways such as p53, Notch, Stat3, MAPK/ERK, hypoxia and the pluripotency marker Nanog did not show any increase over cells in GM **(S4 Table)**. Collectively, these data demonstrated that the LVA captured the dynamics of activation of several Pr/RE representing various pathways and SMC genes during myogenic differentiation of MSCs.

### Use of LVDP reporters to monitor the effects of signaling pathways on MSC myogenic differentiation

Next we monitored the responses of Pr/RE when the signaling network is perturbed. To this end, we used well-known chemical inhibitors targeting the TGF-β1 receptor, Rho/ROCK, extracellular signal-regulated protein kinase (ERK), p38 kinase or c-JUN N-terminal kinase (JNK) pathways and monitored the responses of CArG-RE (SMC transcription) and ACTA2-Pr (early SMC marker). The response was compared to the protein levels of αSMA, the quintessential marker of SMC phenotype, the levels of which have been correlated with SMC contractile function by prior studies [44, 45].

First, hHF-MSCs were transduced with LVDP carrying the aforementioned Pr/RE and three days later, the indicated pathways were blocked by treatment with chemical inhibitors in the presence of DM. Cells cultured in DM or GM without inhibitors served as positive and negative control, respectively. RFI and GFI were determined every 12 hr for a period of 7 days. The normalized intensities were scaled such that at time t = 0 hr, intensities in all 3 culture conditions are 0 and the intensity in DM at the last time point is 1 (**see** Materials & Methods **).**

**Fig 6:**
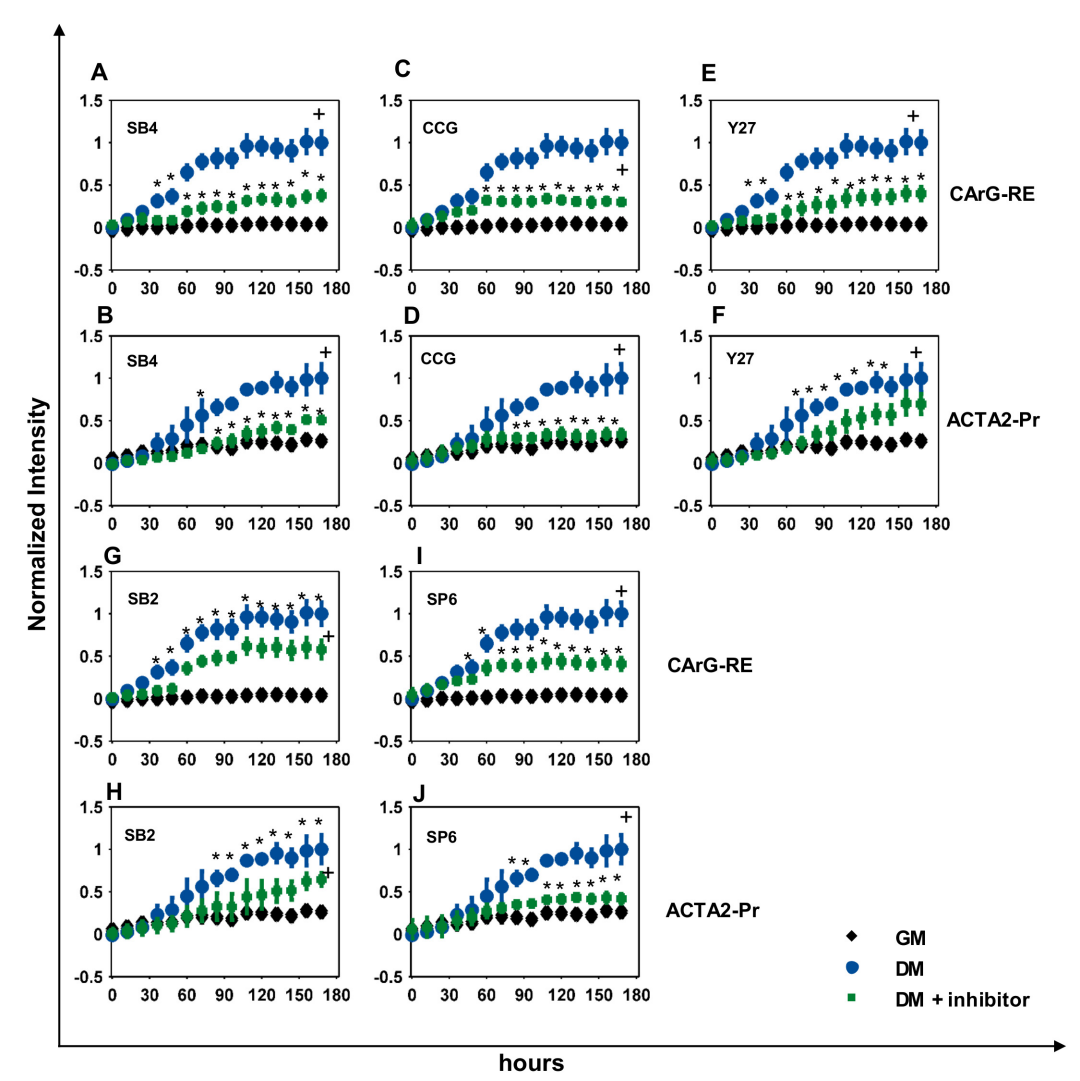
Effects of signaling pathways on Pr/RE activity. Dynamics of Pr/RE activity in hHF-MSCs cultured in DM containing chemical inhibitors. (**A, B)** 10 μM SB431542 (SB4); (**C, D**) 10 μM CCG1423 (CCG); (**E, F**) 10 μM Y27632 (Y27); (**G, H**) 20 μM SB203580 (SB2); (**I, J**) 10 μM SP600125 (SP6). MSCs cultured in GM served as negative control and MSCs cultured in DM served as positive control. Normalized activities of **(A, C, E, G, I)** CArG-RE, and **(B, D, F, H, J)** ACTA2-Pr are shown. The normalized values were scaled from 0–1 and plotted as a function of time. * indicates p < 0.05 between DM and GM as determined by Student’s two-tailed *t*-test at individual time points. + indicates statistical significance of the Pr/RE activities under DM vs GM evaluated over the entire curve by growth curve analysis (p < 0.05).

**Fig 7:**
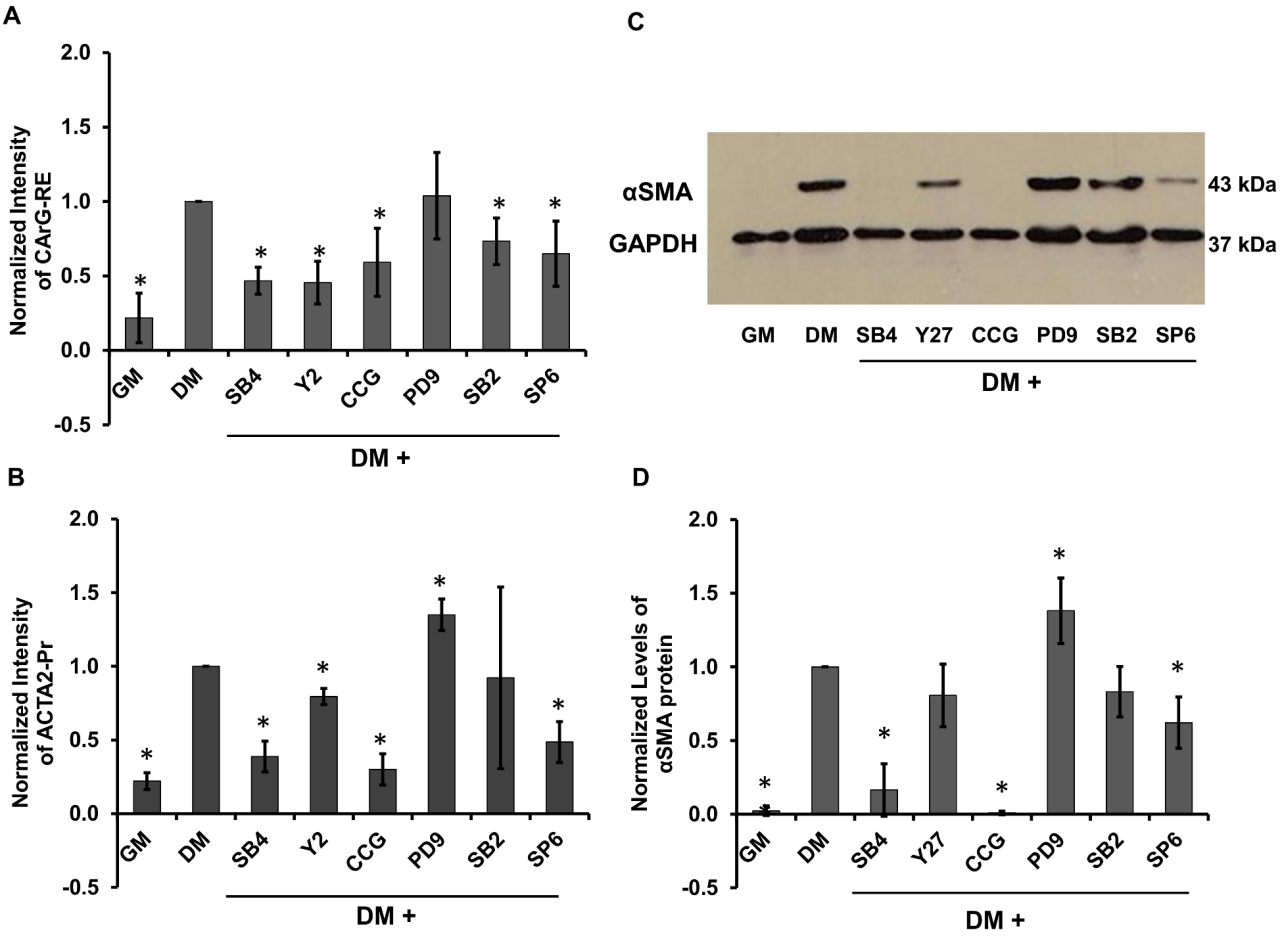
Effects of pathway inhibition on CArG-RE and ACTA2-Pr activity and αSMA protein levels. Response of **(A)** CArG-RE and **(B)** ACTA2-Pr on day 7 of differentiation in the presence of the indicated inhibitors. **(C)** Western blot for αSMA on day 7 of differentiation in the presence of the indicated inhibitors. GAPDH served as loading control. **(D)** Relative expression of αSMA as determined from the band intensities in **(C)**. Normalized intensities were scaled to the intensity in DM without inhibitors. * denotes statistical significance (p<0.05, n = 3) as compared to DM.

Interestingly, SB431542 (SB4) blocked CArG-RE significantly but not completely (NI_SB4_ = 0.47 ± 0.09) (**Figs 6A & 7A)**. The response of ACTA2-Pr was suppressed up to day 4 and reached to only ~40% of its response in DM by day 7 (NI_SB4_ = 0.38 ± 0.1) (**Fig 6B & Fig 7B)**. In agreement with promoter activity, αSMA protein levels were also suppressed significantly down to about ~16% of the level in DM (NI_SB4_ = 0.16 ± 0.17) (**Fig 7C & 7D)**. As expected, blocking the TGF-β signaling pathway by SB4 completely suppressed the activity of SMAD2/3-RE (NI_SB4_ = 0.44 ± 0.13, NI_GM_ = 0.33 ± 0.16) and SMAD7-RE to basal levels (NI_SB4_ = 0.28 ± 0.04, NI_GM_ = 0.23 ± 0.09) (**S1 Fig)**.

Inhibiting the Rho pathway by CCG1423 (CCG) affected the activity of CArG-RE only after day 3, when it prevented CArG-RE activity from increasing above 60% of DM (NI_CCG_ = 0.59 ± 0.22) (**Figs 6C & 7A)**. On the other hand, RhoA inhibition significantly blocked the ACTA2-Pr activity to near basal levels (NI_CCG_ = 0.3 ± 0.1, NI_GM_ = 0.22 ± 0.05) **(Fig 6D & Fig 7B)** and completely suppressed αSMA protein levels **(Fig 7C & 7D)**. Inhibition of ROCK pathway by Y27632 (Y27) partially suppressed the CArG-RE activity (NI_Y27_ = 0.45 ± 0.14) (**Fig 6E & Fig 7A)**. Surprisingly, the activity of ACTA2-Pr was only slightly decreased by ROCK inhibition and reached ~80% of its response in DM (NI_Y27_ = 0.79 ± 0.05) (**Fig 6F & Fig 7B)**. This was also verified by western blot at the protein level (NI_Y27_ = 0.80 ± 0.21) (**Fig 7C & 7D)**.

Among the other signaling pathways, inhibiting the p38 pathway by SB203580 (SB2) suppressed the CArG-RE only slightly by ~27% (NI_SB2_ = 0.73 ± 0.15) (**Fig 6G & Fig 7A)**. ACTA2-Pr expression was similar to its basal levels up to day 4 but gradually increased to 0.92 ± 0.61 over the next 3 days (**Fig 6H & Fig 7B)** in agreement with the αSMA protein level (NI_SB2_ = 0.83 ± 0.17) **(Fig 7C & 7D)**. Inhibition of JNK pathway by SP600125 (SP6) did not affect CArG-RE for the first ~60 hr, after which SP6 kept the CARG-RE response to ~65% of the DM (NI_SP6_ = 0.65 ± 0.21), suggesting that JNK might be important for myogenic differentiation at later times (**Fig 6I & Fig 7A)**. ACTA2-Pr activity was however significantly suppressed by JNK inhibition (NI_SP6_ = 0.48 ± 0.13) (**Fig 6J & Fig 7B)**, which was also corroborated by western blot for αSMA (NI_SP6_ = 0.62 ± 0.17) (**Fig 7C & 7D)**. Finally, ERK inhibition by PD98059 (PD9) only slightly increased the ACTA2-Pr activity (NI_PD9_ = 1.35 ± 0.1) **(Fig 7B)** and did not affect CArG-RE **(Fig 7A)**. The protein levels of αSMA also showed a small increase by ~30% (NI_PD9_ = 1.38 ± 0.22) **(Fig 7C & 7D)**.

Taken together, these data demonstrated the CArG-RE activity in response to the inhibitors tested was in agreement with αSMA protein levels, albeit it was affected to a lesser extent. On the other hand, the ACTA2-Pr activity mirrors the αSMA protein levels under all conditions tested, thereby suggesting that the ACTA2-Pr activity may provide an excellent reporter to monitor the transition of MSC to SMC phenotype.

## Discussion

Differentiation involves transcriptional regulation of a number of genes that take place for a period of several days or weeks. Methods that enable real-time monitoring of transcriptional changes and activation of signaling pathways as stem cells change phenotype provide dynamic information of the differentiation process. In this study we presented a rapid, non-destructive method for high throughput quantitative measurements of gene and pathway activation in real-time as MSCs were coaxed to differentiate into SMCs. We have previously shown that LVDP can be used for quantitative assessment of Pr/RE that are activated in response to inflammatory cytokines [19] as well as the differentiation of MSC into fat, bone or cartilage [22]. Herein we developed a new array of 27 LVDP to study the differentiation of human MSCs from two anatomic locations, namely bone marrow and hair follicle along the SMC lineage. The LVA affords quantitative and dynamic monitoring of live stem cell differentiation for a period of several days and may enable small molecule screening and discovery of genes or pathways affecting lineage specification.

To this end, we employed a lentiviral vector that encodes for two gene cassettes from two independent promoters. This vector has been previously designed in our laboratory to eliminate promoter interference, thereby enabling quantitative measurements of promoter activity independent of the gene transfer efficiency i.e. the number of gene copies per cell [18]. One reporter, ZsGreen is expressed from the Pr/RE of interest, i.e. either the promoter of a SMC-specific gene or the RE that reveals activation of a signaling pathway. The other reporter, DRE2 is expressed from a constitutive promoter, enabling normalization to render the data independent of the efficiency of gene transfer. Indeed, our data shows that increasing the virus titer increased GFI and RFI but the normalized promoter activity remained unchanged. The importance of normalization has also been demonstrated in transfection based assays. In these studies normalization of bioluminescence signals allowed for quantitative comparison of transcription factor activity when exposed to various stimulants [13, 14].

One of the challenges we faced in choosing a normalizing strategy was that both GFI and RFI exhibited heteroscedasticity. To address this issue, we used a data driven approach for data normalization. We normalized the GFI at each time point with a weighted average of RFI over time; the weights were determined from a beta distribution such that the median standard deviation of the normalized intensities was minimized from the LOESS fit. This approach not only stabilized the variance of the normalized data but also significantly improved reproducibility between replicates.

**Fig 8:**
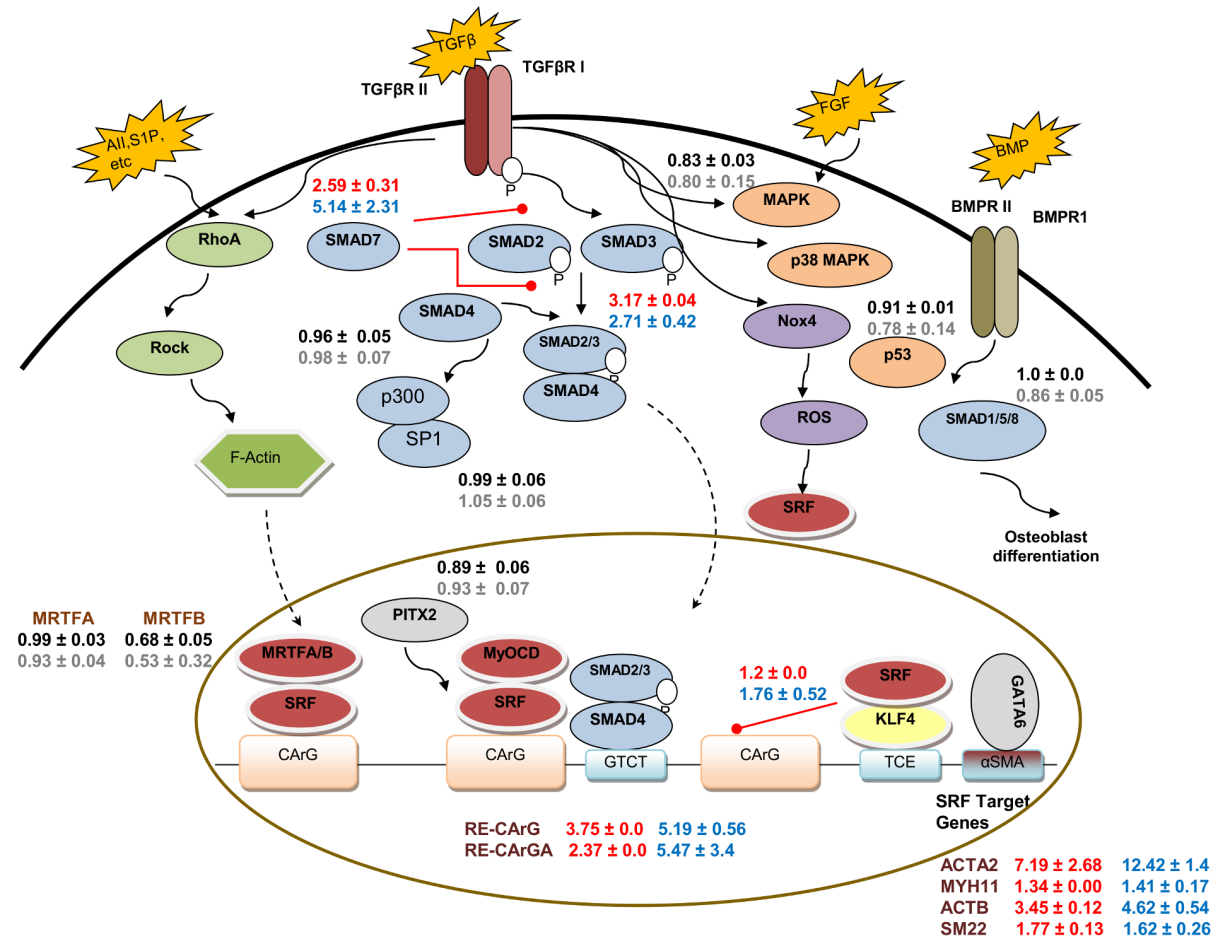
Signaling pathways during myogenic differentiation of MSCs. Signaling pathways during SMC differentiation were compiled from literature [2, 31, 32, 47–50]. External factors (TGFβ, S1P etc) bind to their respective cell surface receptors and activate the downstream signaling pathways that translocate the transcription factors into the nucleus, which then activate the SMC markers. Solid arrows indicate the signaling to the downstream targets in the cytoplasm. Dotted arrows indicate nuclear translocation. Red lines with solid circle end represent inhibition of activity of the target molecule. Normalized fold changes (NI_DM/GM_) for Pr/RE in hBM-MSCs were mapped onto the pathway. Red and blue font indicates significant up-regulation in hBM-MSC and hHF-MSC, respectively while red font indicates no significant response in DM in hBM-MSC and hHF-MSC, respectively. AII–Angiotensin II; S1P-Sphingosine-1-phosphate; BMP–Bone Morphogenetic Protein; BMPRII–Bone morphogenetic protein receptor II; TGFβ–Transforming Growth Factor β; TGFβR II–Type II Transforming Growth Factor β Receptor; FGF–Fibroblast Growth Factor; P–Phosphorylation. Gene names and descriptions are provided in S6 Table.

From the dynamic LVA experiments, we found that 9 out of 27 Pr/RE were significantly active during myogenic differentiation in both hHF-MSCs and hBM-MSCs. These included Smads and SMC specific genes in agreement with previous reports [23, 31, 46]. The molecules that were activated during differentiation and the fold change observed for each Pr/RE in hBM-MSC and hHF-MSC were depicted in a schematic showing the signaling pathways where each of them is known to participate (**Fig 8**). The classic signaling cascade downstream of TGF-β involves activation of the Smad family of transcription factors [47]. Treatment with TGF-β1 phosphorylates Smad2/3, which then binds to Smad4 forming a complex that enters the nucleus and activates Smad responsive promoters. On the other hand, inhibitory Smads, Smad6/7, inhibit the TGF-β signaling forming a negative feedback loop [47]. Indeed, among all the Smad response elements that we tested, Smad2/3-RE and Smad7-RE were activated by TGF-β1, whereas neither Smad1/5/8-RE, a target of the bone morphogenetic protein (BMP) signaling, nor Smad4 showed any change in activity for either type of MSC. Interestingly, similar to Smad2/3-RE, Smad7-RE showed a significant and rapid response in both MSCs, suggesting that the negative feedback loop may be triggered as soon as the TGF-β signaling is activated. As expected, inhibiting TGF-β signaling blocked the activity of Smad2/3-RE and Smad7-RE, while inhibiting other signaling pathways did not significantly affect the Smad-REs (data not shown). Collectively, these results suggest that the Smad-REs can capture the dynamics of TGF-β1 signaling, which are ultimately responsible for development of the SMC phenotype.

TGF-β dependent SMC-specific gene transcription is regulated by SRF, which binds to the CArG cis elements that are found in the promoters of almost all SMC specific genes [31, 48, 51, 52]. In particular, expression of αSMA is induced by binding of SRF to two highly conserved CArG boxes, designated A and B as well as the TGF-β control element (TCE) [43]. Indeed, we observed a quick and significant induction of both CArG-RE and CArGA-RE activity in both hBM-MSCs and hHF-MSCs upon TGF-β1 stimulation. On the other hand, Kruppel-like Factor 4 (KLF4/GKLF) is known to bind to the TCE and negatively regulate SMC gene expression but its response to TGF-β1 is controversial. Some studies reported that TGF-β down-regulated KLF4 [53], while others reported that TGF-β induced the expression of Kruppel-like factors, regardless of their role in regulation of SMC gene expression [54]. Interestingly, we observed a moderate increase in KLF4-RE in hHF-MSCs but no change in hBM-MSCs, suggesting that regulation of KLF4 by TGF-β1 might be context dependent and might vary among MSCs originating from different anatomic locations.

During SMC differentiation certain genes are expressed in temporal order starting with the early genes (ACTA2) and followed by intermediate (SM22) and late genes (MYH11) [55]. Interestingly, our dynamic data not only showed significant promoter activities of ACTA2, SM22 and rMYH11 but also captured the sequential order of their activation. Among other SRF target genes, the ACTB promoter showed a significant change upon TGF-β stimulation, while others e.g. Desmin (DES) and Cysteine and glycine-rich protein 2 (CSRP2) remained unchanged. One possible explanation is that these genes may be already highly expressed and therefore, TGF-β may not trigger further increase in their expression.

We also examined whether the ACTA2-Pr activity reflected the protein levels of αSMA under conditions that inhibited the well-known Smad and non-Smad signaling pathways during myogenic differentiation of hHF-MSCs. Not surprisingly, inhibition of TGF-β1 signaling significantly reduced the ACTA2-Pr activity as well as αSMA protein levels. However, the CArG-RE activity was only partially blocked, suggesting that SRF binding to CArG boxes might be at least partially mediated through non-Smad signaling pathways. Inhibiting p38 had no effect on ACTA2-Pr or αSMA protein but had a small effect on CArG-RE, while inhibition of MAPK/ERK had a small positive effect of ACTA2-Pr and αSMA but no effect on CArG-RE activity. These results suggest that p38 may have a small effect on SRF target genes and that MAPK/ERK might inhibit TGF-β1-induced αSMA expression, albeit it to a small extent as well. Interestingly, the CArG-RE and ACTA2-Pr activities as well the αSMA level were suppressed by inhibition of JNK, in agreement with previous studies implicating JNK as a mediator of TGF-β signaling in fibroblasts, myofibroblasts and hepatic stellate cells [56–59].

Among the non-Smad signaling pathways, Rho/ROCK signaling has been shown to be important in SMC differentiation [31, 48, 60]. CCG1423, a Rho/MRTF/SRF pathway inhibitor, significantly reduced the ACTA2-Pr activity as well as the level of αSMA, possibly by blocking the MRTFA translocation to the nucleus, where it regulates SMC specific gene expression in association with SRF [61]. On the other hand, inhibiting ROCK decreased ACTA2-Pr activity at early times and to a lesser extent at late times, in agreement with previous studies showing that inhibition of ROCK suppressed SMC gene expression but did not inhibit it completely [31, 60, 62]. Interestingly, the CArG-RE activity decreased significantly upon ROCK inhibition, suggesting that while ACTA2 expression might be regulated mostly through the Rho/MRTF/SRF pathway, other SRF target genes might be also regulated by ROCK.

It would also be interesting to investigate the role of other non-Smad signaling pathways such as the Notch signaling in SMC differentiation of MSC. Notch signaling has been shown to promote SMC differentiation, however its effects likely depend on the cell type. The deletion of the Notch ligand Jagged 1 (JAG1) has been shown to repress the expression of SMC marker genes in hBM-MSCs [63]. Several studies have shown that inhibiting the notch intracellular domain inhibits SMC differentiation while its overexpression stimulates SMC marker gene expression [31]. However the downstream signaling of SMC differentiation via Notch signaling is unknown and will need further investigation. Our novel LVDP can be used in conjunction with chemical inhibitors of Notch signaling to understand how it influences SMC differentiation. Alternatively, key molecules in the Notch signaling can be knocked down using a novel shRNA encoding LVDP that our group has developed previously [64].

In summary, our results demonstrate that the LVA can be used to monitor the dynamics of gene and pathway activation during stem cell differentiation into SMC. The transcriptional kinetics captured the sequential activation of signaling pathways and SMC specific genes. Selected reporters also captured the effects of biochemical pathways on SMC protein levels, suggesting that in combination with chemical libraries, LV reporters may be used to delineate the role of signaling pathways or identify novel inducers or inhibitors of lineage specification. Similarly, in combination with siRNA libraries, they may be used to identify novel genes that may be involved in stem cell differentiation. It may also be possible to employ such quantitative, dynamic data from large-scale LVA to reverse engineer gene networks that govern stem cell fate decisions or other cellular processes. Finally, LV reporters may be particularly useful in single cell studies where use of traditional assays e.g. RT-PCR or western blots may be difficult or may require sophisticated microfabrication technologies, which may not be widely available.

## Supporting Information

S1 Fig Effects of blocking TGF-β1 on Smad-RE activity. Dynamics of (**A)** Smad2/3-RE and (**B)** Smad7-RE activity in hHF-MSCs cultured in DM and 10 μM SB431542 (SB4). MSCs cultured in GM served as negative control and MSCs cultured in DM served as positive control. The normalized values were scaled from 0–1 and plotted as a function of time. * indicates p < 0.05 between DM + SB4 and DM as determined by Student’s two-tailed *t*-test at individual time points. + indicates statistical significance of the Pr/RE activities under DM vs DM + SB4 evaluated over entire curve by growth curve analysis (p < 0.05).
(TIFF)

S1 Table Oligos and primers used for cloning transcriptional response elements and promoters, respectively, in the LVDP. (PDF)

S2 Table List of Chemical Inhibitors. (PDF)

S3 Table Details of datasets analyzed. (PDF)

S4 Table Fold changes of Promoters and Response Elements. (PDF)

S5 Table Promoter and Response Element Activation. (PDF)

S6 Table List of Genes. (PDF)
