# Supplementary figures and images for: Lentivirus Live Cell Array for Quantitative Assessment of Gene and Pathway Activation during Myogenic Differentiation of Mesenchymal Stem Cells
Source: PLoS One. 2015 Oct 27;10(10):e0141365. doi: 10.1371/journal.pone.0141365 (PMC4624764; doi:10.1371/journal.pone.0141365)

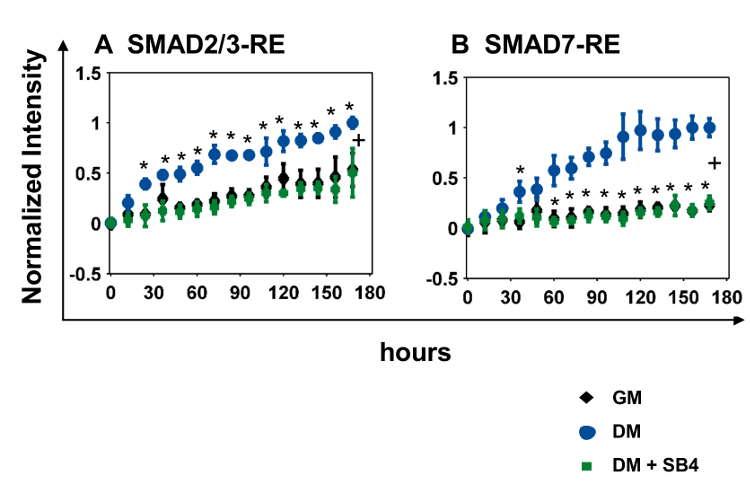

Supplement: S1 Fig — Dynamics of (A) Smad2/3-RE and (B) Smad7-RE activity in hHF-MSCs cultured in DM and 10 μM SB431542 (SB4). MSCs cultured in GM served as negative control and MSCs cultured in DM served as positive control. The normalized values were scaled from 0–1 and plotted as a function of time. * indicates p < 0.05 between DM + SB4 and DM as determined by Student’s two-tailed t-test at individual time points. + indicates statistical significance of the Pr/RE activities under DM vs DM + SB4 evaluated over entire curve by growth curve analysis (p < 0.05). (TIFF) [file pone.0141365.s001.tiff]
