# Supplementary material for: Lentivirus Live Cell Array for Quantitative Assessment of Gene and Pathway Activation during Myogenic Differentiation of Mesenchymal Stem Cells
Source: PLoS One. 2015 Oct 27;10(10):e0141365. doi: 10.1371/journal.pone.0141365 (PMC4624764; doi:10.1371/journal.pone.0141365)
Supplement: S1 Table — (PDF) [file pone.0141365.s002.pdf]

S1 Table. Oligos and primers used for cloning transcriptional response elements and promoters, respectively, in the LVDP

REs	*Cloning Sites*	*Oligos/Primers (5' to 3')*	*Reference*
MAPK/ERK-RE	BstBI, HpaI	For: AACGGATGTCCATATTAGGAGGATGTCCATATTAGGAGGATGTCCATATT AGGAGGATGTCCATATTAGGAGGATGTCCATATTAGGATT<br>Rev: CGAATCCTAATATGGACATCCTCCTAATATGGACATCCTCCTAATATGGA CATCCTCCTAATATGGACATCCTCCTAATATGGACATCCGTT	SABiosciences, inc.
CArG-RE	BstBI, HpaI	For: AACCCATAAAAGGCCATAAAAGGCCATAAAAGGCCATAAAAGGCCATAA AAGGCCATAAAAGGTT<br>Rev: CGAACCTTTTATGGCCTTTTATGGCCTTTTATGGCCTTTTATGGCCTTTTAT GGCCTTTTATGGGTT	SABiosciences, inc.
CArGA-RE	BstBI, HpaI	For: AACCCTTGTTTGGCCTTGTTTGGCCTTGTTTGGCCTTGTTTGGCCTTGTTTG GCCTTGTTTGGTT<br>Rev: CGAACCAAACAAGGCCAAACAAGGCCAAACAAGGCCAAACAAGGCCAA ACAAGGCCAAACAAGGGTT	(1)
EGR1-RE	BstBI, HpaI	For: AACCGCCCCCGCGCGCCCCCGCGCGCCCCCGCGCGCCCCCGCGCGCCCCC GCGCGCCCCCGCGTT<br>Rev: CGAACGCGGGGGCGCGCGGGGGCGCGCGGGGGCGCGCGGGGGCGCGCG GGGGCGCGCGGGGGCGGTT	SABiosciences, inc.
KLF4-RE	BstBI, HpaI	For: AACAGGGTGTGGCCAGGGTGTGGCCAGGGTGTGGCCAGGGTGTGGCCAG GGTGTGGCCAGGGTGTGGCCTT<br>Rev: CGAAGGCCACACCCTGGCCACACCCTGGCCACACCCTGGCCACACCCTGG CCACACCCTGGCCACACCCTGTT	SABiosciences, inc.
MEF2-RE	BstBI, BsiWI	For: GTACGCTAGCGCTCTAAAAATAACCCTCTAGCGCTCTAAAAATAACCCTC TAGCGCTCTAAAAATAACCCTCTAGCGCTCTAAAAATAACCCTTTT<br>Rev: CGAAAGGGTTATTTTTAGAGCGCTAGAGGGTTATTTTTAGAGCGCTAGAG GGTTATTTTTAGAGCGCTAGAGGGTTATTTTTAGAGCGCTAGC	(2)
SP1-RE	BstBI, BsiWI	For: GTACGGGGGCGGGGCGGGGCGGGGCGGGGCGGGGCGGGGCGGGGCGGG GCGGGGCGGGGCGGGGCTT<br>Rev: CGAAGCCCCGCCCCGCCCCGCCCCGCCCCGCCCCGCCCCGCCCCGCCCCG CCCCGCCCCGCCCCC	SABiosciences, inc.
ATF6-RE	BstBI, BsiWI	For: GTACGCTCGAGACAGGTGCTGACGTGGCATTCCTCGAGACAGGTGCTGAC GTGGCATTCCTCGAGACAGGTGCTGACGTGGCATTCTT<br>Rev: CGAAGAATGCCACGTCAGCACCTGTCTCGAGGAATGCCACGTCAGCACCT GTCTCGAGGAATGCCACGTCAGCACCTGTCTCGAGC	SABiosciences, inc.

HIF1-RE	BstBI, BsiWI	For: GTACGTACGTGCTTACGTGCTTACGTGCTTACGTGCTTACGTGCTTACGTGCTTT<br>Rev: CGAAAGCACGTAAGCACGTAAGCACGTAAGCACGTAAGCACGTAAGCACGTAC	SABiosciences, inc.
Nanog-RE	BstBI, BsiWI	For: GTACGACCCTTCGCCGATTAAGTACTTAAACCCTTCGCCGATTAAGTACTTAAACCCTTCGCCGATTAAGTACTTAATT<br>Rev: CGAATTAAGTACTTAATCGGCGAAGGGTTTAAGTACTTAATCGGCGAAGGGTTTAAGTACTTAATCGGCGAAGGGTC	SABiosciences, inc.
STAT3-RE	BstBI, BsiWI	For: GTACGGTCGACATTTCCCGTAAATCGTCGAGTCGACATTTCCCGTAAATCGTCGAGTCGACATTTCCCGTAAATCGTCGATT<br>Rev: CGAATCGACGATTTACGGGAAATGTCGACTCGACGATTTACGGGAAATGTCGACTCGACGATTTACGGGAAATGTCGACC	SABiosciences, inc.
p53-RE	BstBI, BsiWI	For: GTACGAGACATGTCCAGACATGTCCGAACATGTCCCAACATGTTGTAGACATGTCCAGACATGTCCGAACATGTCCCAACATGTTGTTT<br>Rev: CGAAACAACATGTTGGGACATGTTCGGACATGTCTGGACATGTCTACAACATGTTGGGACATGTTCGGACATGTCTGGACATGTCTC	SABiosciences, inc.
SMAD2/3-RE	BstBI, BsiWI	For: GTACGAGCCAGACAAGCCAGACAAGCCAGACAAGCCAGACAAGCCAGACAAGCCAGACATT<br>Rev: CGAATGTCTGGCTTGTCTGGCTTGTCTGGCTTGTCTGGCTTGTCTGGCTTGTCTGGCTC	SABiosciences, inc.
SMAD4-RE	BstBI, BsiWI	For:GTACGGGCGCGGGCGCGGGCGCGGGCGCGGGCGCGGGCGCGGGCGCGTT<br>Rev:CGAACGCGCCCGCGCCCGCGCCCGCGCCCGCGCCCGCGCCCGCGCCC	SABiosciences, inc
SMAD7-RE	BstBI, BsiWI	For: GTACGTAGCCAGACAGTAGCCAGACAGTAGCCAGACAGTAGCCAGACAGTAGCCAGACAGTAGCCAGACAGTAGCCAGACAGTT<br>Rev: CGAACTGTCTGGCTACTGTCTGGCTACTGTCTGGCTACTGTCTGGCTACTGTCTGGCTACTGTCTGGCTACTGTCTGGCTAC	(3)
SMAD1/5/8-RE	BstBI, BsiWI	For: FOR: CACAGTTAACGAGCTAACATAACCCGGGAGGT<br>Rev: CACATTCGAAACCAACAGTACCGGAATGCCAAGC	Provided by Dr. Blobe (4)
Notch-RE	BstBI, HpaI	For: AACCGTGGGAACGTGGGAACGTGGGAACGTGGGAACGTGGGAACGTGGGAATT<br>Rev: CGAATTCCCACGTTCCCACGTTCCCACGTTCCCACGTTCCCACGTTCCCACGGTT	SABiosciences, inc.
ACTA2-Pr	EcoRI, AgeI	For: ACAACAGGATCCCTTCCTGTCAAGGAGGTTAG<br>Rev: ACAACAACCGGTCGGGTAATTAAAAGAGCCAC	(5)
SM22-Pr	EcoRI, AgeI	For: ACAACAGGATCCGGCCTGGTGTGGAGTAGGTC<br>Rev: ACAACAACCGGTGGTTCGGACAGACAGGATGG	(6)
SMTNB-Pr	EcoRI, AgeI	For: ACAACAGGATCCGGCCTCTGGCACTGAAGATG<br>Rev: ACAACAACCGGTGGATTCTGCGGAGAGCTGCC	(7)
MKL1-Pr	ClaI, AgeI	For: ACAACAATCGATCGGGCAAGGGTATAGGATTT<br>Rev: ACAACAACCGGTAACCAAGAGGGTGGGAAAGA	http://www.switchdb.com/

PITX2-Pr	ClaI, AgeI	For: ACAACAATCGATCGTGATCTGAGGCGACTTTT<br>Rev: ACAACAACCGGTAAGAGTGACAGGGACAGGTA	(8)
DES-Pr	EcoRI, ClaI	For: ACAACAGAATTCTTTATGGGGTCAGGTAGTTG<br>Rev: ACAACAATCGATGTTGAGAGATTCAATTCTGC	http://www.switchdb.com/
CSRP2-Pr	ClaI, AgeI	For: ACAACAATCGATGAGGTCATGAGGAGGCCTAG<br>Rev: ACAACAACCGGTCCTACTCACTTGAGTCGGAG	http://www.switchdb.com/
MKL2-Pr	ClaI, AgeI	For: ACAACAATCGATTTGGGAAGTTTGTAAAGTGA<br>Rev: ACAACAACCGGTGACGAACAGGGATGGCTGCC	http://www.switchdb.com/
ACTB-Pr	ClaI, AgeI	For: ACAACAATCGATATAGCAGACATACAACGGAC<br>Rev: ACAACAACCGGTCAAAGGCGAGGCTCTGTGCT	http://www.switchdb.com/
rMYH11-Pr (rabbit)	ClaI, AgeI	For: ACAACAATCGATTCCACACTCAGCCATCTCAC<br>Rev: ACAACAACCGGTAGCAAGAGCTCCACAACTCA	(9)

Supplemental References:

1. Hautmann MB, Madsen CS, Owens GK. A transforming growth factor beta (TGFbeta) control element drives TGFbeta-induced stimulation of smooth muscle alpha-actin gene expression in concert with two CArG elements. J Biol Chem. 1997;272(16):10948-56.
2. Lu J, McKinsey TA, Nicol RL, Olson EN. Signal-dependent activation of the MEF2 transcription factor by dissociation from histone deacetylases. Proc Natl Acad Sci U S A. 2000;97(8):4070-5.
3. Bajpai VK, Mistriotis P, Loh YH, Daley GQ, Andreadis ST. Functional vascular smooth muscle cells derived from human induced pluripotent stem cells via mesenchymal stem cell intermediates. Cardiovasc Res. 2012;96(3):391-400.
4. Bajpai VK, Mistriotis P, Andreadis ST. Clonal multipotency and effect of long-term in vitro expansion on differentiation potential of human hair follicle derived mesenchymal stem cells. Stem Cell Res. 2012;8(1):74-84.
5. Reddy S, Ozgur K, Lu M, Chang W, Mohan SR, Kumar CC, et al. Structure of the human smooth muscle alpha-actin gene. Analysis of a cDNA and 5' upstream region. J Biol Chem. 1990;265(3):1683-7.
6. Solway J, Seltzer J, Samaha FF, Kim S, Alger LE, Niu Q, et al. Structure and expression of a smooth muscle cell-specific gene, SM22 alpha. J Biol Chem. 1995;270(22):13460-9.
7. Rensen SS, Thijssen VL, De Vries CJ, Doevendans PA, Detera-Wadleigh SD, Van Eys GJ. Expression of the smoothelin gene is mediated by alternative promoters. Cardiovasc Res. 2002;55(4):850-63.
8. Kioussi C, Briata P, Baek SH, Rose DW, Hamblet NS, Herman T, et al. Identification of a Wnt/Dvl/beta-Catenin --> Pitx2 pathway mediating cell-type-specific proliferation during development. Cell. 2002;111(5):673-85.
9. Katoh Y, Loukianov E, Kopras E, Zilberman A, Periasamy M. Identification of functional promoter elements in the rabbit smooth muscle myosin heavy chain gene. J Biol Chem. 1994;269(48):30538-45.
