# Supplementary material for: Lentivirus Live Cell Array for Quantitative Assessment of Gene and Pathway Activation during Myogenic Differentiation of Mesenchymal Stem Cells
Source: PLoS One. 2015 Oct 27;10(10):e0141365. doi: 10.1371/journal.pone.0141365 (PMC4624764; doi:10.1371/journal.pone.0141365)
Supplement: S2 Table — (PDF) [file pone.0141365.s003.pdf]

S2 Table. List of Chemical Inhibitors

Inhibitor	*Pathway*	*Concentration*	*Inhibitor Target*	*Company*
SB431542	TGFβ1	10 µM	ALK4/5/7: TGFβ-R1	Tocris
Y27632	ROCK	10 µM	ROCK1	Cayman Chemical
CCG1423	RhoA	10 µM	Interaction of SRF with MKL1	Cayman Chemical
SB203580	p38	20 µM	p38 mapk	Cayman Chemical
PD98059	ERK	10 µM	MEK1	Enzo Life Sciences
SP600125	JNK	10 µM	JNK1/2/3	Cayman Chemical
