# Supplementary material for: Lentivirus Live Cell Array for Quantitative Assessment of Gene and Pathway Activation during Myogenic Differentiation of Mesenchymal Stem Cells
Source: PLoS One. 2015 Oct 27;10(10):e0141365. doi: 10.1371/journal.pone.0141365 (PMC4624764; doi:10.1371/journal.pone.0141365)
Supplement: S3 Table — (PDF) [file pone.0141365.s004.pdf]

S3 Table. Details of datasets analyzed

Parameter	*hBM-MSC*	*hHF-MSC*	*Chemical Inhibitor Screen (hHF-MSC)*
G	2	3	3
H	27	27	4
I	2 (GM, DM)	2 (GM, DM)	10 (GM, DM & 8 inhibitors)
J	4	4	4
K	18	22	15
Shape parameters, beta distribution	(3.41,1.48)	(0.54,0.11)	(1,0.377)
