# Supplementary material for: Lentivirus Live Cell Array for Quantitative Assessment of Gene and Pathway Activation during Myogenic Differentiation of Mesenchymal Stem Cells
Source: PLoS One. 2015 Oct 27;10(10):e0141365. doi: 10.1371/journal.pone.0141365 (PMC4624764; doi:10.1371/journal.pone.0141365)
Supplement: S4 Table — (PDF) [file pone.0141365.s005.pdf]

**S4 Table. Fold changes of Promoters and Response Elements**

| *hBM-MSC (n=2)* | | | *hHF-MSC (n=3)* | | |
|---|---|---|---|---|---|
| *Pr/RE* | *Fold change* | *p-value (curve)* | *Pr/RE* | *Fold change* | *p-value (curve)* |
| **ACTA2-Pr** | **7.19 ± 2.68** | **4.65E-07** | **ACTA2-Pr** | **12.42 ± 1.40** | **1.23E-08** |
| **CArG-RE** | **3.75 ± 0.00** | **1.95E-04** | **CArGA-RE** | **5.47 ± 3.40** | **7.07E-06** |
| **ACTB-Pr** | **3.45 ± 0.12** | **9.24E-06** | **CArG-RE** | **5.19 ± 0.56** | **3.00E-03** |
| **Smad2/3-RE** | **3.17 ± 0.04** | **3.31E-07** | **Smad 7-RE** | **5.14 ± 2.31** | **3.35E-06** |
| **Smad7-RE** | **2.59 ± 0.31** | **7.65E-06** | **ACTB-Pr** | **4.62 ± 0.54** | **5.99E-06** |
| **CArGA-RE** | **2.37 ± 0.00** | **4.17E-07** | **Smad2/3-RE** | **2.71 ± 0.42** | **4.77E-06** |
| **SM22-Pr** | **1.77 ± 0.13** | **7.94E-07** | **KLF4-RE** | **1.76 ± 0.52** | **1.85E-02** |
| **rMYH11-Pr** | **1.34 ± 0.00** | **5.26E-04** | **SM22-Pr** | **1.62 ± 0.26** | **2.74E-02** |
| **KLF4-RE** | **1.20 ± 0.00** | **1.50E-02** | **rMYH11-Pr** | **1.41 ± 0.17** | **9.07E-03** |
| ATF6-RE | 2.31 ± 0.05 | 2.16E-03 | HIF1-RE | 1.91 ± 1.13 | 8.12E-01 |
| STAT3-RE | 1.36 ± 0.01 | 5.26E-01 | KLF5-Pr | 1.75 ± 0.56 | 6.72E-01 |
| KLF5-Pr | 1.11 ± 0.48 | 5.50E-01 | ATF6-RE | 1.63 ± 1.00 | 7.83E-01 |
| DES-Pr | 1.07 ± 0.03 | 4.42E-02 | DES-Pr | 1.26 ± 0.21 | 1.20E-02 |
| EGR1-RE | 1.05 ± 0.11 | 5.69E-02 | EGR1-RE | 1.07 ± 0.02 | 2.65E-01 |
| SMAD1/5/8-RE | 1.00 ± 0.00 | 7.53E-03 | SP1-RE | 1.05 ± 0.06 | 2.47E-01 |
| HIF1-RE | 1.02 ± 0.04 | 4.66E-02 | CSRP2-Pr | 1.03 ± 0.10 | 9.31E-02 |
| SMTNB-Pr | 0.99 ± 0.05 | 2.57E-02 | Smad4-RE | 0.98 ± 0.07 | 1.54E-01 |
| MKL1-Pr | 0.99 ± 0.03 | 4.96E-02 | SMTNB-Pr | 0.98 ± 0.09 | 1.00E-03 |
| SP1-RE | 0.99 ± 0.06 | 2.60E-01 | Nanog-RE | 0.90 ± 0.26 | 8.82E-01 |
| CSRP2-Pr | 0.98 ± 0.01 | 5.00E-03 | MKL1-Pr | 0.93 ± 0.04 | 2.14E-01 |
| Smad4-RE | 0.96 ± 0.05 | 1.70E-03 | PITX2-Pr | 0.93 ± 0.07 | 2.64E-02 |
| p53-RE | 0.91 ± 0.01 | 1.99E-02 | SMAD1/5/8-RE | 0.86 ± 0.05 | 2.81E-01 |
| PITX2-Pr | 0.89 ± 0.06 | 1.52E-01 | ERK-RE | 0.80 ± 0.15 | 4.92E-02 |
| ERK-RE | 0.83 ± 0.03 | 2.52E-03 | p53-RE | 0.78 ± 0.14 | 3.05E-01 |
| Nanog-RE | 0.83 ± 0.09 | 4.62E-01 | STAT3-RE | 0.75 ± 0.20 | 6.20E-02 |
| MEF2-RE | 0.82 ± 0.07 | 1.26E-03 | MEF2-RE | 0.59 ± 0.11 | 3.00E-04 |
| MKL2-Pr | 0.68 ± 0.05 | 4.70E-02 | MKL2-Pr | 0.53 ± 0.32 | 4.16E-01 |
