# Supplementary material for: Lentivirus Live Cell Array for Quantitative Assessment of Gene and Pathway Activation during Myogenic Differentiation of Mesenchymal Stem Cells
Source: PLoS One. 2015 Oct 27;10(10):e0141365. doi: 10.1371/journal.pone.0141365 (PMC4624764; doi:10.1371/journal.pone.0141365)
Supplement: S5 Table — (PDF) [file pone.0141365.s006.pdf]

S5 Table. Promoters and Response Element Activation

Pr/RE	*Time of activation (hours)*	
	hBM-MSC (n=2)	*hHF-MSC (n=3)*
**SMAD2/3-RE**	**20.00 ± 5.66**	**45.33 ± 12.22**
**SMAD7-RE**	**16.00 ± 0.00**	**21.33 ± 4.62**
**CArG-RE**	**20.00 ± 5.66**	**21.33 ± 4.62**
**CArGA-RE**	**32.00 ± 0.00**	**61.33 ± 9.24**
**KLF4-RE**	**116.00 ± 16.97**	**80.00 ± 16.00**
**ACTA2-Pr**	**20.00 ± 5.66**	**40.00 ± 8.00**
**SM22-Pr**	**52.00 ± 5.65**	**64.00 ± 8.00**
**rMYH11-Pr**	**52.00 ± 5.65**	**92.00 ± 16.97**
**ACTB-Pr**	**48.00 ± 0.00**	**37.33 ± 12.22**
