# Supplementary material for: Lentivirus Live Cell Array for Quantitative Assessment of Gene and Pathway Activation during Myogenic Differentiation of Mesenchymal Stem Cells
Source: PLoS One. 2015 Oct 27;10(10):e0141365. doi: 10.1371/journal.pone.0141365 (PMC4624764; doi:10.1371/journal.pone.0141365)
Supplement: S6 Table — (PDF) [file pone.0141365.s007.pdf]

S6 Table. List of Genes

UniGene Symbol	*UniGene Name*
ACTA2	Actin, alpha 2, smooth muscle, aorta
ACTB	Actin, Beta
ATF6	Activating transcription factor 6
CNN1	Calponin 1
CSRP2	Cysteine and glycine-rich protein 2
DES	Desmin
EGR1	Early growth response 1
HIF1	Hypoxia inducible factor 1
KLF4	Kruppel-like factor 4
MEF2	Myocyte Enhancer Factor 2
MKL1	Megakaryoblastic leukemia (translocation) 1, Myocardin-related transcription factor A (MRTFA)
MKL2	Megakaryoblastic leukemia (translocation) 2, Myocardin-related transcription factor B (MRTFB)
MYH11	Myosin, Heavy Chain 11, Smooth Muscle
PITX2	Paired-like homeodomain 2
SM22	Transgelin
SMTNB	Smoothelin B
SP1	Sp1 transcription factor
STAT3	Signal transducer and activator of transcription 3
VCL	Vinculin
